# Very high prevalence of 25-hydroxyvitamin D deficiency in 6433 UK South Asian adults: analysis of the UK Biobank Cohort

**DOI:** 10.1017/S0007114520002779

**Published:** 2021-02-28

**Authors:** Andrea L. Darling, David J. Blackbourn, Kourosh R. Ahmadi, Susan A. Lanham-New

**Affiliations:** School of Biosciences and Medicine, Faculty of Health and Medical Sciences, University of Surrey, Guildford GU2 7XH, UK

**Keywords:** 25-Hydroxyvitamin D, Ethnicity, Epidemiology, Cohort studies

## Abstract

Little research has assessed serum 25-hydroxyvitamin D (25(OH)D) concentration and its predictors in Western-dwelling South Asians in a relatively large sample size. This observational, cross-sectional analysis assessed baseline prevalence of 25(OH)D deficiency in UK-dwelling South Asians (aged 40–69 years, 2006–2010) from the UK Biobank Cohort. Serum 25(OH)D measurements were undertaken using the DiaSorin Liaison XL assay. Of 6433 South Asians with a 25(OH)D measurement, using commonly used cut-off thresholds, 55 % (*n* 3538) had 25(OH)D < 25 nmol/l (severe deficiency) and 92 % (*n* 5918) had 25(OH)D < 50 nmol/l (insufficiency). Of the participants with a measurement, 20 % (*n* 1287) had 25(OH)D concentration <15 nmol/l (very severe deficiency). When 824 participants with undetectable (<10 nmol/l) 25(OH)D measurements were included (total *n* 7257), 29 % (*n* 2105) had 25(OH)D < 15 nmol/l, 60 % (*n* 4354) had 25(OH)D < 25 nmol/l and 93 % (*n* 6749) had 25(OH)D < 50 nmol/l. Logistic regression predictors of 25(OH)D < 25 nmol/l included the following characteristics: being male; Pakistani; higher BMI; 40–59 years old; never consuming oily fish; summer sun exposure <5 h/d, not using a vitamin D-containing supplement, measurement in winter or spring and vegetarianism. In terms of region, median 25(OH)D concentration was 19–20 nmol/l in Scotland, Northern England, the Midlands and Wales. Across Southern England and London, it was slightly higher at 24–25 nmol/l. Our analyses suggest the need for increased awareness of vitamin D deficiency in South Asians as well as urgent public health interventions to prevent and treat vitamin D deficiency in this group.

A high prevalence of 25-hydroxyvitamin D (25(OH)D) deficiency in Western-dwelling South Asians in countries with a relatively high latitude has been found, for example, in Europe^(1)^ and Canada^(2)^. Postulated reasons for 25(OH)D deficiency in this population group include low vitamin D intake from diet^(3)^ as well as low usage of vitamin D-containing supplements^(3)^, a covered dress style and some sun avoidance^(4)^.

In the UK, some small-scale research has examined 25(OH)D concentration in South Asian populations and there is the need for larger-scale research. For example, research from the UK Diet, Food Intake, Nutrition and Exposure to Sunlight in Southern England (D-FINES) study found that 54 % of premenopausal South Asian women studied had severe deficiency (25(OH)D < 25 nmol/l), which rose to 81 % in winter^(5)^. Equivalent values for insufficient 25(OH)D concentration (<50 nmol/l) were 95–96 % depending on season^(5)^. Data from the same study for postmenopausal women showed that 51 % of South Asian women had 25(OH)D < 25 nmol/l in summer, rising to 65 % of women having 25(OH)D < 25 nmol/l in winter^(6)^.

In Northern England and the Midlands, prevalence of vitamin D deficiency is also very high. For example, a study of South Asian men and women living in Manchester (53·5°N) found that >90 % had 25(OH)D concentration <25 nmol/l in winter, with 100 % <50 nmol/l^(7)^. In two studies from the Midlands, South Asian men and women had an average 25(OH)D concentration of 25–33 nmol/l^(8,9)^. Of note, in Birmingham, Shaunak *et al*. found a mean 25(OH)D of only 21 nmol/l in British Hindu Asian men and women, with 22 % having very severe deficiency (25(OH)D < 10 nmol/l)^(10)^.

Clearly, throughout England, as in other Western countries^(1,2,4)^, deficiency in 25(OH)D is epidemic in South Asian adults, with many having severe vitamin D deficiency. However, in the UK, there is a lack of data for South Asians living in Northern Ireland, Scotland and Wales, as well as a general lack of data in children and adolescents. Indeed, only one study, to the authors’ knowledge, has assessed 25(OH)D concentration in UK South Asian children and found that in 618 South Asian children, 20–34 % had 25(OH)D concentration <25 nmol/l^(11)^. Moreover, as mentioned above, sample sizes in the studies that have been conducted to date have been relatively small. Few South Asians are included in national diet and health surveys, meaning that there are to date no large-scale assessments of 25(OH)D status in South Asian populations living in either the UK or in other Western countries. Further research into 25(OH)D status of this population group is needed from larger sample sizes.

The UK Biobank Cohort, which covers England, Scotland and Wales, has the largest dataset globally, to the authors’ knowledge, reporting 25(OH)D concentrations in Western-dwelling South Asian adults. Participants were aged 40–69 years at baseline, and the South Asian sub-cohort has 8024 individuals (of which, *n* 6433 have valid data for 25(OH)D concentration). This sample size provides a great opportunity to study 25(OH)D concentration, and its predictors, with a relatively large sample size, including providing information on 25(OH)D concentration in South Asians living in Scotland and Wales. We report here the main findings of 25(OH)D status in this cohort, assessing predictive factors such as sex, ethnic sub-group (Bangladeshi, Indian, Pakistani), season of measurement, dietary and lifestyle factors, anthropometry and geographical region.

## Methods

### UK Biobank Cohort

The UK Biobank is a cohort of 500 000 individuals, aged 40–69 years at recruitment, of which 8024 are of self-reported ethnicity as South Asians (defined as Bangladeshi, Indian or Pakistani). Of these, 6433 have valid baseline data for serum 25(OH)D concentration. Data on vitamin D-containing supplement usage and vitamin D intakes in this South Asian sub-cohort have been published previously^(3)^. This analysis is an observational study with cross-sectional design, analysing data from the UK Biobank Cohort baseline measurement (taken during the period 2006–2010). Participants were recruited via invitation through NHS central patient registers^(12)^. Participants were from across the UK, in England, Scotland and Wales, living at latitudes ranging from Glasgow and Edinburgh in Scotland to Reading in the South. No assessment centres were present in the East of England, the North of Scotland, the South West or Northern Ireland.

### Measurement of 25-hydroxyvitamin D concentration

Throughout this paper and its Supplementary file, 25(OH)D concentration represents total 25(OH)D (i.e. both 25(OH)D_2_ and 25(OH)D_3_ combined) as data were not reported for these two metabolites separately by UK Biobank. Blood draws were spread across the year with each individual attending once. Participants were not fasted. Serum 25(OH)D measurements were produced using the DiaSorin Liaison XL assay, which is a direct competitive chemiluminescent immunoassay that measures both 25(OH)D_2_ and 25(OH)D_3_. Therefore, the 25(OH)D measurement reflects vitamin D_2_ (a plant and fungi source of vitamin D) as well as vitamin D_3_ (derived from photosynthesis in the skin and from animal sources). Of note, this assay underestimates 25(OH)D by 4 % at 25 nmol/l, but overestimates 25(OH)D by 5–10 % at ≥40 nmol/l^(13)^. The lower limit of detection is 10 nmol/l. This should be borne in mind when interpreting the results presented in this paper. Full details of the assay quality-control procedures, including precision, accuracy and bias as well as linearity (between-lot) and multi-instrument comparisons, as implemented by UK Biobank can be found in the UK Biobank documentation^(14)^.

### Analysis

#### Descriptives

All analyses were conducted using SPSS v25 software. Figures were created using GraphPad Prism v7.02 software. For defining serum 25(OH)D deficiency, we used the commonly used three cut-points: <25 nmol/l (deficiency)^(15)^; <50 nmol/l (insufficiency)^(16,17)^ and 75 nmol/l (optimal)^(18)^. Although not widely used, the cut-off of 15 nmol/l was also chosen for inclusion in descriptive analysis as we considered an additional cut-off below 25 nmol/l to be useful to our analyses. This was because a large number of the participants were below 25 nmol/l.

The primary outcome of the study was 25(OH)D concentration, including both median (interquartile range, IQR) and percentage of persons under different 25(OH)D cut-offs. To investigate possible bias in the results caused by 824 individuals not having a 25(OH)D reading due to their sample being below readable limits (<10 nmol/l), a sub-analysis was conducted whereby these individuals were assigned the commonly used correction of lower limit/square root of 2 as their 25(OH)D reading (see online Supplementary Tables S1–S3). No other procedures were put in place to compensate for missing data in the main analysis.

Serum 25(OH)D was not normally distributed and so non-parametric tests were used to assess statistical significance (log transformation failed to normalise the variable). Regarding analysis of outliers, no 25(OH)D values were removed from the dataset as all were <300 nmol/l and therefore were considered feasible concentrations. Only baseline variables were used with one exception. For vitamin D intake only, follow-up measures as well as baseline vitamin D intakes were used in order to more accurately assess vitamin D intake as well as increase the number of participants with vitamin D intake data.

#### Coding of dietary variables – descriptives

dietary variables, details of the UK Biobank 24-h recall and dietary FFQ(touchscreen) have been previously published^(3)^. Briefly, the 24-h recall questionnaire was computer touchscreen based and asked for frequency of different foods consumed in the last 24 h, using a FFQ (touchscreen) called the Oxford Web Q FFQ^(19)^. This questionnaire produced the data for vitamin D intakes (combined vitamins D_2_ and D_3_, excluding supplement use). See previous publication^(3)^ for details of number of participants and numbers of dietary assessment follow-ups available.

General consumption of food from different dietary categories was assessed via a different touchscreen FFQ. As in our previous paper^(3)^, we used this questionnaire to identify vegetarians (defined as those who ate no meat or fish) by including those who answered ‘never’ to the following categories: oily fish, non-oily fish, poultry, beef, lamb and mutton, pork and processed meat. Similarly, the oily fish question on this questionnaire was used to measure oily fish consumption. Participants originally recorded oily fish consumption as ‘never, less than once per week, once per week, 2–4 times per week, 5–6 times per week and once or more daily’. In this analysis, due to low subject numbers having oily fish five times per week or more, this was reclassified as ‘never, less than once per week, once per week and two or more times per week’.

Vitamin D-containing supplement use was defined as per our previous paper^(3)^, being those who consumed either a single vitamin D supplement, a multivitamin and mineral supplement or both. We were not able to assess dosage, brand or supplement use frequency as participants were not asked to give this information. It was not possible to analyse usage of cod liver oil as the Biobank only asked about fish oil supplements in general (it did not differentiate cod liver oil from *n*-3 fish oils).

#### Coding of non-dietary variables

For non-dietary variables, all were self-reported except for assessment centre, 25(OH)D concentration, blood draw date and time, BMI and waist:hip ratio which were assessed and reported by Biobank staff. Townsend deprivation index score was calculated by Biobank staff based on self-reported postcode (zip code). See Supplementary file for full details of the original coding, as well as any recoding, for non-dietary variables undertaken for this analysis.

#### Power calculation

In terms of a power calculation, we used the following published data on 25(OH)D concentration in South Asian men and women. For South Asian adults living in Ottawa (45°N) (aged 20–79 years), men had mean 25(OH)D 44·9 (sd 17·2) nmol/l and women had 25(OH)D 54·8 (sd 28·5) nmol/l^(20)^. Using these data, a power calculation for sex differences in 25(OH)D, assuming a mean difference of 10 nmol/l and a sd of 23 nmol/l, a sample size of 112 men and 112 women are required for 90 % power, *α* = 0·05. With 6433 (3506 men and 2927 women), the study is sufficiently powered for this analysis and in fact is likely to be overpowered. Therefore, in logistic regression models, 95 % CI were used (rather than *P* values) for interpreting statistical significance.

#### Logistic regression

In the logistic regression models, the first category was set to be the reference category. 25(OH)D was dummy coded as <25 nmol/l or ≥25 nmol/l. There were too few South Asians over 50 nmol/l (6 %) to conduct the same analysis with 50 nmol/l as the cut-off point. Variables were categorised as previously described in the Methods section and in the Supplementary file. A wide array of predictors were included, including anthropometric (e.g. BMI), demographic (e.g. household income, Townsend deprivation index), dietary factors (e.g. vitamin D supplement use), medications (e.g. statin usage) and factors related to the blood draw (e.g. season). In terms of sub-analyses, the logistic regression model was also conducted in each sub-ethnicity and sex separately in order to assess the predictors that are important for each of these groups.

#### Ethical approval

The UK Biobank study was conducted according to the guidelines laid down in the Declaration of Helsinki, and all procedures involving human subjects were approved by the UK North West Multi-Centre Research Ethics Committee (MREC); application 11/NW/0382. Written informed consent was obtained from all subjects.

## Results

### Descriptives

**Fig. 1.**
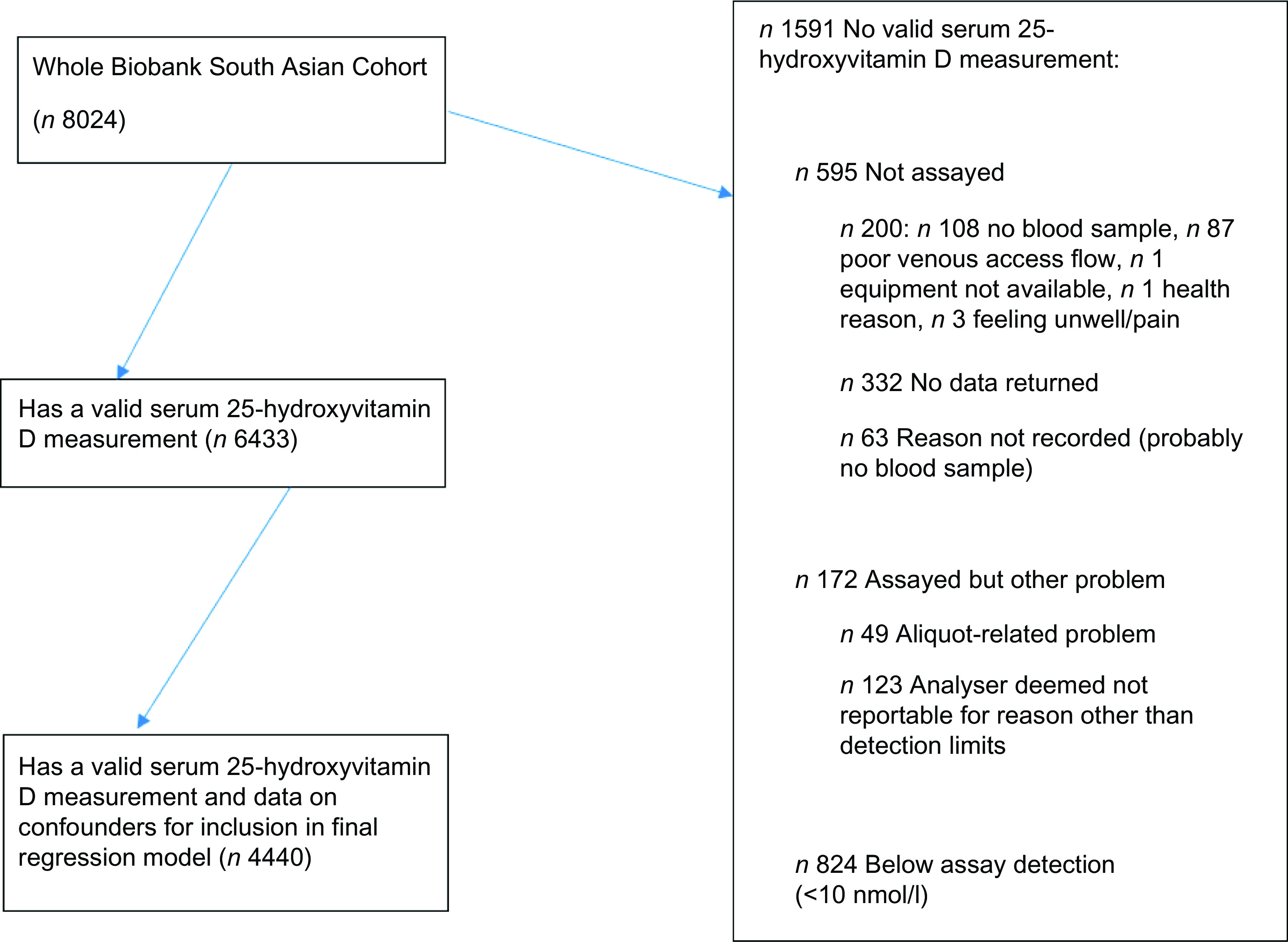
Flow diagram to illustrate flow of participants from original South Asian subset of the UK Biobank (*n* 8024) to those in the current analysis (*n* 6433) and specifically the logistic regression model (*n* 4440).

Of the 8024 South Asians, 6433 had a 25(OH)D measurement. See online Supplementary file and Fig. 1 for information regarding the reasons for missing data in 1591 participants. See Table 1 for detail of participant characteristics for the included participants in the analysis (*n* 6433), by ethnic sub-group and sex.

**Table 1.** Categorical descriptives (only those who have a valid vitamin D measurement; *n* 6433) (Percentages)

	All SA (*n* 6433)*	Bangladeshi (*n* 207)	Indian (*n* 4792)	Pakistani (*n* 1434)	Men (*n* 3506)	Women (*n* 2927)
Season						
Spring	24·7	25·6	24·2	26·1	25·0	24·3
Summer	35·1	27·1	36·6	31·0	34·0	36·4
Autumn	24·1	23·2	24·3	23·4	24·0	24·2
Winter	16·2	24·2	14·9	19·5	17·1	15·2
Missing	0	–	–	–	–	–
*P*	–	<0·0001			0·09	
Age						
40–60 years	71·7	80·2	68·9	79·8	70·5	73·0
>60 years	28·3	19·8	31·1	20·2	29·5	27·0
Missing	0	–	–	–	–	–
*P*	–	<0·001			0·03	
Income						
<£18 000	23·1	61·3	25·5	45·8	30·6	31·5
£18 000 to £30 900	18·0	13·1	25·4	21·2	23·9	24·5
£31 000 to £51 900	15·4	16·1	22·1	16·2	20·0	21·5
≥£52 000	18·1	9·5	26·9	16·8	25·5	22·5
Missing	25·4	–	–	–	–	–
*P*	–	<0·001			0·11	
BMI (kg/m^2^)						
≤25·4	32·3	40·5	35·4	24·1	32·6	33·3
26–29·4	44·4	44·0	45·1	46·6	48·5	41·6
≥30	21·1	15·5	19·6	29·3	18·9	24·8
Missing	2·1	–	–	–	–	–
*P*		<0·001			<0·001	
Region						
North England	22·2	27·1	16·7	40·2	24·4	19·6
South England	8·0	4·8	8·5	6·8	7·7	8·4
Wales	1·4	2·4	1·1	2·0	1·5	1·3
Scotland	2·2	1·9	1·6	4·1	2·5	1·9
Midlands	19·9	17·9	19·6	21·3	21·1	18·5
Greater London	46·3	45·9	52·5	25·6	42·9	50·3
Missing	0	**–**	**–**	**–**	–	–
*P*	**–**	<0·001	–	–	<0·001	
Oily fish consumption						
Never	30·2	3·5	35·4	20·8	24·8	38·7
<Once per week	28·0	17·5	26·5	38·8	32·0	25·2
Once per week	27·1	27·5	27·0	31·6	29·8	25·9
≥2 times per week	11·6	51·5	11·2	8·8	13·4	10·2
Missing	3·2	–	–	–	–	–
*P*		<0·001			<0·001	
Born UK/ROI						
No	87·3	95·4	89·5	87·3	90·1	88·1
Yes	10·6	4·6	10·5	12·7	9·9	11·9
Missing	2·1	–	–	–	–	–
*P*	–	0·001			0·01	
Vegetarian						
Yes	16·8	0·5	27·4	0·7	12·9	27·6
No	68·1	99·5	72·6	99·3	87·1	72·4
Missing	15·1					
*P*		<0·001			<0·001	
Vitamin D-containing supplement†						
Yes	23·8	16·6	25·8	20·9	18·7	31·3
No	73·6	83·4	74·2	79·1	81·3	68·7
Missing	2·6					
*P*	–	<0·001			<0·001	
Self-reported health						
Good/excellent	56·9	43·6	60·9	49·4	58·7	56·8
Fair/poor	41·5	56·4	39·1	50·6	41·3	43·2
Missing	1·6					
*P*	–	<0·001			0·13	
Oral contraceptive use (females)						
Yes	0·8	3·0	1·8	1·6	–	–
No	43·7	97·0	98·2	98·4	–	–
Missing	44·5				–	–
*P*		0·73			–	–
Use of cholesterol-lowering medication						
Yes	26·3	39·0	26·9	27·7	33·4	20·6
No	69·6	61·0	73·1	72·3	66·6	79·4
Missing	4·1					
*P*		<0·001			<0·001	
Time spent outdoors (h)						
<1	10·8	16·7	11·9	11·1	9·4	15·0
1–2	36·2	36·1	40·4	38·5	37·3	43·0
3–4	24·9	30·6	27·7	25·9	28·1	26·4
≥5	18·9	16·7	20·0	24·5	25·2	15·5
Missing	9·2	–	–	–	–	–
*P*	–	0·005	–	–	<0·001	
Current smoker						
No	90·2	73·4	92·9	87·7	86·2	97·0
Yes	8·8	26·6	7·1	12·3	13·8	3·0
Missing	1·1					
*P*	–	<0·001			<0·001	
Skin tone						
Very fair/fair	16·1	14·8	15·0	22·2	14·4	19·2
Light or dark olive	17·7	15·8	16·8	23·0	12·5	24·9
Brown or black	63·7	69·4	68·3	54·9	73·1	56·0
Missing	2·5					
*P*	–	<0·001			<0·001	
Use of sun protection						
Never/rarely	45·7	61·2	44·2	60·7	56·3	38·8
Sometimes	33·2	29·8	37·6	27·5	32·8	38·1
Most of time/always	15·5	9·0	18·1	11·7	10·9	23·1
Missing	5·6					
*P*	–	<0·001			<0·001	

SA, South Asian, ROI, Republic of Ireland. All *P* values are *χ* ^2^.

* Percent including missing data, all other percentages are valid percent (i.e. excluding missing data).

† Vitamin D-containing supplement means either single vitamin D supplement or multivitamin which contains vitamin D.

#### Categorical descriptives – general

For categorical descriptives, Bonferroni adjustment with a revised *P* value of (*P* < 0·001) was used to ascertain statistical significance due to the large number of statistical tests (*n* 35) conducted on these background characteristics. Variables of some statistical and biological relevance are listed in Table 1 and those of minimal statistical and biological relevance are listed in online Supplementary Table S4. All the associations reported here were statistically significant at *P* < 0·001.

#### Categorical descriptives – age, season and country of birth

In terms of ethnicity and season of blood draw, a higher percentage of the Indian group had their draw in the summer (36·6 %) than did the Bangladeshi (27·1 %) and Pakistani (31 %) groups. Accordingly, the Indian group had less blood draws in the winter.

There were small ethnic differences in age, with 10–11 % more Indians in the older age category (>60 years) than Bangladeshis or Pakistanis. Bangladeshis had a lower income than the other two groups, with 36 % more Bangladeshis than Indians, as well as 16 % more Bangladeshis than Pakistanis, in the lowest income bracket (<£18 k per year). There was an association between ethnic group and being born outside the UK/Republic of Ireland. The Pakistani and Indian groups had similar percentages of participants who were born outside the UK/Republic of Ireland (87–90 %), Bangladeshis, however, were slightly more likely to have been born outside the UK/Republic of Ireland (95 %).

#### Categorical descriptives – region, obesity and health

There was an association between geographical region and ethnicity, with the modal category for Pakistanis being Northern England, compared with Greater London for Bangladeshis and Indians. For sex, differences in geographical region were present but relatively small (<1 to 7 %, depending on region), with the largest sex differences in Northern England (5 % less women) and Greater London (7 % less men). There was also an association between ethnic group and BMI, with Pakistanis having the highest prevalence of overweight/obesity (of 25 kg/m^2^ or greater) (76 %), followed by Indians (65 %) and Bangladeshis (60 %). There was an association between BMI and sex, with more persons with obesity (BMI of 30 kg/m^2^ or greater) in women (25 %) than in men (19 %). There was an association between ethnicity and self-reported health. Less Indians reported only fair/poor health (39 %) than did Bangladeshis (56 %) and Pakistanis (51 %), so self-reported health was better in the Indian group.

#### Categorical descriptives – diet

In terms of diet, there was an association between oily fish consumption and ethnicity, with 52 % of Bangladeshis consuming oily fish two or more times per week, compared with only 9 % of Pakistanis and 11 % of Indians. Similarly, there was an association between oily fish consumption and sex, with slightly more men (13 %) eating oily fish two or more times per week than did women (10 %), and concurrently, more women (39 %) than men (25 %) reported that they never ate oily fish. Vegetarianism varied by ethnic group, with more Indians being vegetarian (27 %) compared with Pakistanis and Bangladeshis (<1 %). Similarly, more women reported being vegetarian (28 %), compared with men (13 %). As also found in our previous paper^(3)^, usage of a vitamin D containing supplement was associated with ethnicity and sex. More Indians reported taking this kind of supplement (26 %) than did Bangladeshis (17 %) or Pakistanis (21 %). Also, more women (31·3 %) took a vitamin D-containing supplement than did men (18·7 %).

#### Categorical descriptives – other factors

Self-reported time spent outdoors in summer varied by sex, with a higher number of women (15 %) spending less than 1 h/d outside than did men (9 %). Indians were less likely to report ‘never or rarely using sun protection’ (44 %) compared with Bangladeshis and Pakistanis (both 61 %). Also, men were more likely to report this (56 %) than were women (39 %). Very few women were using oral contraceptives (only 3 %), and this usage was not associated with ethnic group. Use of cholesterol-lowering medications varied by both sex and ethnic group, being higher in men (33 %) compared with women (21 %), as well as being higher in Bangladeshis (39 %) compared with Indians and Pakistanis (27–28 %). Pakistanis were slightly less likely to report brown or black skin tone (54·9 %) than were Bangladeshis (69 %) and Indians (68 %). Women were less likely to report brown or black skin tone (56 %) than were men (73 %).

Finally, smoking status varied by ethnicity, with a higher percentage of Indians (93 %) being non-smokers compared with Pakistanis (88 %) or Bangladeshis (73 %). Similarly, a higher percentage of women were non-smokers (97 %) compared with men (86 %).

#### Continuous variables

Table 2 illustrates median and IQR data for continuous variables by ethnic group and sex. Bonferroni adjustment with a revised *P* value of (*P* < 0·005) was used to ascertain statistical significance due to the number of statistical tests (*n* 10) conducted on these background characteristics. Median waist:hip ratio varied by sex but not ethnic group, with women having a slightly smaller waist:hip ratio (0·85 (IQR 0·1)) compared with men (0·95 (IQR 0·1)) (*P* < 0·001). This was higher than the 0·88 for men and 0·81 for women recommended for health in South Asian population^(21)^. Townsend deprivation index varied between ethnic groups (*P* < 0·001), with Bangladeshis having the most deprived score (3·5), which was higher than Pakistanis (1·4) and Indians (–0·3).

**Table 2.** Continuous variables by ethnic group and sex (*n* 6433) (Median values and interquartile ranges (IQR); numbers)

	All SA (*n* 6433)			Bangladeshi (*n* 207)			Indian (*n* 4792)			Pakistani (*n* 1434)			*P* *
	Median	IQR	*n*	Median	IQR	*n*	Median	IQR	*n*	Median	IQR	*n*	
Fasting time (h)	4·0	2·0	6433	4·0	2·0	207	4·0	2·0	4792	4·0	2·0	1434	0·007
BMI (kg/m^2^)	26·6	5·2	6295	25·8	5·2	203	26·3	5·0	4677	27·6	5·5	1415	<0·001
Waist:hip ratio	0·91	0·11	6380	0·93	0·1	203	0·90	0·1	4762	0·93	0·1	1415	<0·001
Vitamin D intake (µg/d)	1·20	1·8	1982	3·2	5·0	34	1·1	1·8	1650	1·5	1·7	298	<0·001
Townsend deprivation index†	0·1	4·6	6425	3·5	6·7	207	–0·3	4·2	4786	1·4	5·1	1432	<0·001

	Men			Women			*P*						
	Median	IQR	*n*	Median	IQR	*n*							
Fasting time (h)	4·0	2·0	3506	4·0	2·0	2927	0·01						
BMI (kg/m^2^)	26·5	2·7	3392	26·8	5·9	2903	0·03						
Waist:hip ratio	0·95	0·1	3475	0·85	0·1	2905	<0·001						
Vitamin D intake (µg/d)	1·3	1·9	1070	1·1	1·8	912	0·08						
Townsend deprivation index†	0·3	5·1	3502	0·0	4·5	2923	<0·001						

SA, South Asian.

* Kruskal–Wallis test for *P* (ethnicity).

† Higher = more deprived.

### Reasons for missing data by sex and ethnicity

#### Those without 25-hydroxyvitamin D data: reason ‘under detection limit’ (*n* 824)

For these people, there was a more even split within sex (49 % women and 51 % men), compared with those in the original sample of 8024 (46 % women and 54 % men). This suggests that women were slightly over-represented here. In terms of ethnicity, 0·6 % were Bangladeshi, 73 % were Indian and 26 % were Pakistani. This compares with the following percentages in the original sample of 8024: 3 % Bangladeshi, 74 % Indian and 23 % Pakistani. This suggests a slight bias in that Bangladeshis were under-represented in those under the detection limit; however, the percentage of Indians and Pakistanis were as would be expected from the percentages in the 8024 cohort.

#### Those without 25-hydroxyvitamin D data: other reasons (*n* 767)

Other reasons included aliquot problems or having no blood draw. For these people, there was a difference within sex (52 % women and 49 % men), compared with those in the original sample of 8024 (47 % women and 54 % men). This suggests that women were over-represented here, compared with the original sample. In terms of ethnicity, 3 % were Bangladeshi, 73 % were Indian and 24 % were Pakistani. This compares with the following percentages in the original sample of 8024: 3 % Bangladeshi, 74 % Indian and 23 % Pakistani. This suggests a no bias in that the percentage of each ethnic group was as would be expected.

### Primary outcomes

#### 25-Hydroxyvitamin D status

**Fig. 2.**
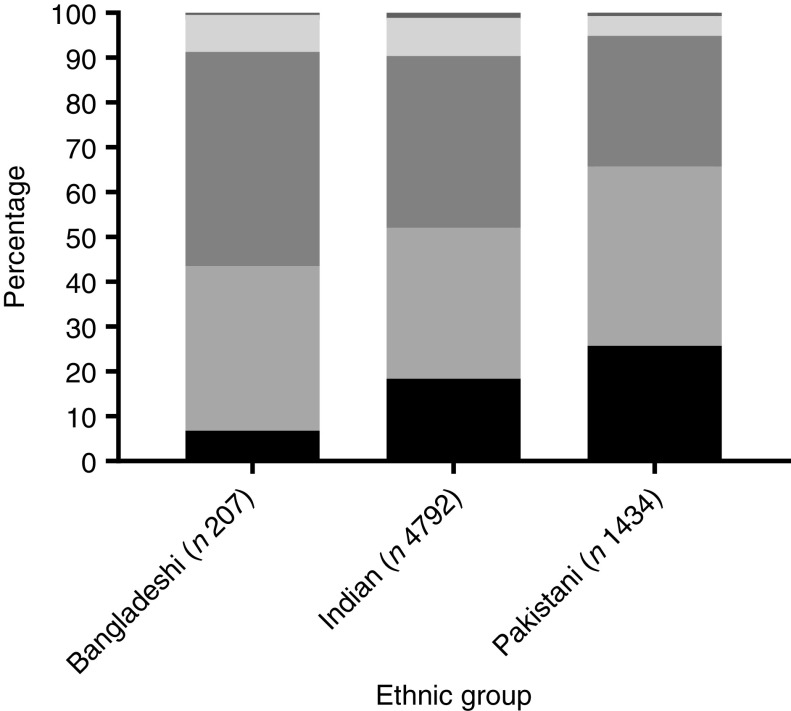
Percentage within each 25-hydroxyvitamin D cut-off category by ethnic group average for the year (all data combined *n* 6433). 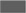, ≥75 nmol/l; 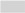, 50–74 nmol/l; 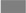, 25–49 nmol/l; 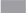, 15–24 nmol/l; 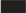, <15 nmol/l.

**Fig. 3.**
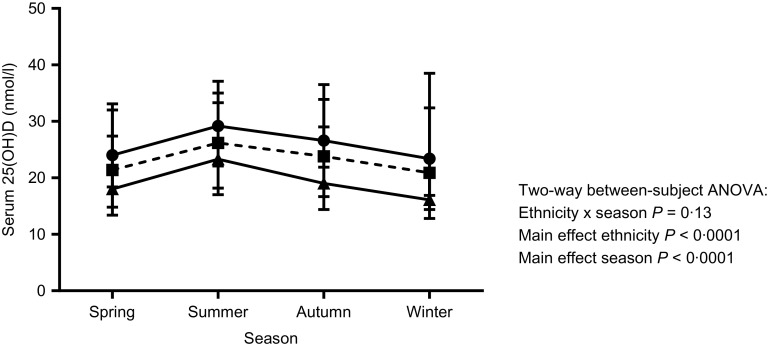
Serum 25-hydroxyvitamin D (25(OH)D) by season and ethnic group (*n* 6433). Each person has one measurement in one season only (data are not repeated measures). Data are medians and interquartile ranges. There was no statistically significant interaction between ethnicity and season, but there was a main effect of ethnicity, with the Bangladeshi group having the highest 25(OH)D in each season and the Pakistani group the lowest. All groups were slightly higher in summer and lower in winter than they were in spring and autumn. 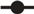, Bangladeshi (*n* 207); 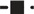, Indian (*n* 4792); 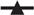, Pakistani (*n* 1434).

Results are given as medians and IQR unless otherwise stated (Table 3). The proportion of persons with 25(OH)D < 15 nmol/l (severe deficiency) and 25(OH)D < 25 nmol/l was high (20 and 55 %, respectively). In addition, nearly all participants had 25(OH)D < 50 nmol/l (92 %) and <75 nmol/l (99 %) (Table 3 and Fig. 2). Regarding ethnic sub-group, Indians and Bangladeshis had a higher median 25(OH)D (24–26 nmol/l) than did Pakistanis (19 nmol/l) (Table 3 and Fig. 3). This ethnic difference in median 25(OH)D is likely only of small biological relevance. However, Bangladeshis had a much lower proportion of people below 15 nmol/l than did Indians or Pakistanis. Men had a slightly lower 25(OH)D (21·7 (IQR 16·2)) than did women (24·3 (IQR 20·5) (Table 3 and online Supplementary Figs. S1 and S2), but this was likely of minimal biological relevance. As can be seen, the IQR is very wide for both sexes, suggesting large variability in 25(OH)D between individuals.

**Table 3.** 25-Hydroxyvitamin D (25(OH)D) concentration as well as percentage of participants below 25(OH)D cut-offs by group and sex (*n* 6433) (Median values and interquartile ranges (IQR); percentages)

Group/25(OH)D (nmol/l)	*n*	Median (nmol/l)	IQR (nmol/l)	<15 nmol/l (%)	<25 nmol/l (%)	<50 nmol/l (%)	<75 nmol/l (%)	*P* value for medians*
All South Asian	6433	22·8	18·1	19·6	54·8	91·5	99·0	
Bangladeshi	207	26·1	14·3	6·8	43·5	91·3	100·0	*P* < 0·0001 ethnic
Indian	4792	23·8	19·3	18·4	52·0	90·4	98·9	
Pakistani	1434	19·3	14·5	25·7	65·7	94·9	99·3	
South Asian men	3506	21·7	16·2	21·1	58·4	93·8	99·5	*P* < 0·0001 sex
South Asian women	2927	24·3	20·5	17·8	50·4	88·6	98·5	

* Mann–Whitney test for South Asian men *v*. South Asian women/Kruskal–Wallis test for Bangladeshi *v*. Indian *v*. Pakistani.

For sex, ethnicity and season, a two way between subjects ANOVA (online Supplementary Table S5) for 25(OH)D concentration showed a sex × season interaction (*P* < 0·0001). Of note, although statistically significant, this interaction would not be considered biologically meaningful, as there was only a 0·2 to 2·4 % sex difference by season (Table 1). For ethnicity and season, there was no interaction (*P* = 0·13), but main effects for both ethnicity and season were statistically significant (*P* < 0·0001). The ethnicity × season interaction is likely to be partly explained by the Indian group having higher proportion of summer blood draws than the other ethnic sub-groups (Table 1).

In terms of geographical region, a Kruskal–Wallis test showed a statistically significant difference in 25(OH)D status between regions, with a latitude gradient present (Scotland lowest at a median of 18·7 (IQR 16·6) nmol/l and London highest at 25·0 (IQR 19·6) nmol/l, online Supplementary Table S6). A clear latitude gradient was seen in the men (Scotland 16·7 nmol/l, Northern England, Midlands and Wales 20 nmol/l and London and Southern England 23–24 nmol/l; *P* < 0·001). The gradient was still present in women, but Scotland was similar to Northern England (Scotland, Northern England, Midlands and Wales 19–21 nmol/l and London and Southern England 25–27 nmol/l, *P* < 0·001).

See the Supplementary file for full details of the sub-analysis of results (online Supplementary Table S1–S3) when including those who had readings below the limit of detection. To briefly summarise, as would be expected, the prevalence of <15 nmol/l rose to 29 % of the whole sample of 7257 (compared with 20 % in the main analysis), with <25 nmol/l rising to a prevalence of 60 % (compared with 55 % in the main analysis).

#### Prediction of 25-hydroxyvitamin D deficiency – logistic regression model

Data are presented as OR and 95 % CI unless otherwise stated. The Townsend deprivation index, household income, geographic region, usage of cholesterol-lowering medications, tanning bed/solarium usage and vitamin D intake were trialled in the model but did not show statistical significance so were removed. Vitamin D deficiency was defined as serum 25(OH)D < 25 nmol/l. The final logistic regression model is shown in Table 4.

**Table 4.** Odds of having <25 nmol/l concentration of 25-hydroxyvitamin D (25(OH)D): logistic regression model including a variety of demographic, anthropometric, dietary- and lifestyle-related variables (Unstandardised *B* coefficients with their standard errors; odds ratios and 95 % confidence intervals)

Model		*n*	*B*	se	OR*	Lower 95 % CI	Upper 95 % CI
Model (*n* 4440) *P* < 0·001 Nagelkerke *R* ^2^ = 0·16	Sex						
	Female	2039			1·00		
	Male	2401	0·25	0·07	1·29	1·13	1·48
	Ethnicity						
	Indian	3222			1·00		
	Pakistani	1072	0·33	0·08	1·39	1·18	1·63
	Bangladeshi	146	–0·54	0·19	0·58	0·41	0·84
	BMI (kg/m^2^)						
	≤25·4 Normal/underweight	1484			1·00		
	26–29·4	2007	0·28	0·07	1·32	1·14	1·53
	≥30	949	0·41	0·09	1·51	1·26	1·80
	Age						
	40–59 years	3139			1·00		
	60 years and over	1301	–0·58	0·07	0·56	0·49	0·65
	Oily fish consumption						
	Never	1590			1·00		
	<Once/week	1227	–0·02	0·10	0·98	0·80	1·20
	Once/week	1168	–0·11	0·10	0·90	0·73	1·10
	2 or more times per week	455	–0·51	0·13	0·60	0·46	0·78
	Daily summer sun						
	<1 h	504			1·00		
	1–2 h	1809	–0·15	0·11	0·86	0·70	1·07
	3–4 h	1207	–0·22	0·12	0·81	0·64	1·01
	≥5 h	920	–0·28	0·12	0·76	0·59	0·96
	Season of blood draw						
	Spring	1093			1·00		
	Summer	1619	–0·71	0·09	0·49	0·42	0·58
	Autumn	1030	–0·31	0·09	0·73	0·61	0·88
	Winter	698	0·09	0·11	1·10	0·89	1·35
	Sun protection usage†						
	Never/rarely	2191			1·00		
	Used sometimes	1554	–0·31	0·07	0·73	0·64	0·84
	Used most of time/always	695	–0·19	0·10	0·83	0·69	1·00
	Vitamin D supplement‡						
	User	1145			1·00		
	Non-user	3295	1·10	0·08	2·99	2·58	3·46
	Vegetarian						
	Yes	905			1·00		
	No	3535	–0·28	0·11	0·75	0·61	0·94
	Constant	0·13	0·15	1·14			

* Odds of having serum 25(OH)D < 25 nmol/l (≥25 nmol/l=reference).

† Usage of sunscreen lotion or hat.

‡ Vitamin D-containing supplement (i.e. single supplement or multivitamin).

Men were 29 % more likely to have vitamin D deficiency (OR 1·29 (95 % CI 1·13, 1·48)) than were women (reference). For ethnicity, compared with Indians (reference category), Pakistanis had an increased odds of deficiency (OR 1·39 (95 % CI 1·18, 1·63)), but Bangladeshis had a reduced odds (OR 0·58 (95 % CI 0·41, 0·84)). For BMI, those who were overweight had a 32 % higher odds of deficiency (OR 1·32 (95 % CI 1·14, 1·53)) and those with obesity had a 51 % higher odds (OR 1·51 (95 % CI 1·26, 1·80)) than did those who were of normal/underweight (reference category). This showed a clear increase in odds of deficiency with increasing BMI category. For age, older persons (60 years or over) had only OR = 0·56 (95 % CI 0·49, 0·65) of the odds of deficiency than did those aged 40–59 years (reference category).

For oily fish consumption, higher intakes were associated with reduced odds of deficiency compared with never consuming oily fish (reference category). Specifically, there was an OR = 0·60 (95 % CI 0·46, 0·78) for oily fish two or more times per week, OR = 0·90 (95 % CI 0·73, 1·10) for once or more per week and OR = 0·98 (95 % CI 0·80, 1·2) for less than once a week. This showed that there was only a reduced odds of deficiency as oily fish consumption increased to two or more times per week. For summer outdoor sunlight exposure, compared with those who reported <1 h/d (reference category), those with 5 h or over daily had reduced odds of deficiency with an OR = 0·76 (95 % CI 0·59, 0·96), but there was no difference for those with 3–4 h (OR = 0·81 (95 % CI 0·64, 1·01)) or 1–2 h (OR = 0·86 (95 % CI 0·70, 1·07)). This shows a reduction in odds with increased summer sunlight exposure over 5 h/d.

Compared with those who had blood drawn in spring (reference category), those with a blood draw in summer were almost half as likely to have deficiency, with an OR = 0·49 (95 % CI 0·42, 0·58), and those with a blood draw in autumn were 27 % less likely to have deficiency, with an OR = 0·73 (95 % CI 0·61, 0·88). Those with a blood draw in winter had no change in odds from that of spring, with an OR = 1·10 (95 % CI 0·89, 1·35). Usage of sun protection (e.g. hat, sun cream) had a reduced odds of deficiency by 27 % if used sometimes (OR 0·73 (95 % CI 0·64, 0·84)) but no reduced odds if used most of the time or always (OR 0·83 (95 % CI 0·69, 1·00)), suggesting a U-shaped (non-linear) relationship between sun protection use and odds of 25(OH)D deficiency. Non-usage of a vitamin D-containing supplement (either single vitamin D or a vitamin D-containing multivitamin) increased odds of deficiency 3-fold, with OR = 3·00 (95 % CI 2·58, 3·46). Finally, not being vegetarian was associated with a reduced odds by 25 % (OR 0·75 (95 % CI 0·61, 0·94)) compared with being vegetarian (reference).

Logistic regression sub-analyses conducting the same model within sex and ethnicity are shown in online Supplementary Tables S7 and S8. To summarise, when running the logistic regression model in the Indian group, sex, BMI, oily fish, sunlight, season and sun protection were significant predictors of vitamin D deficiency. Equivalent results for the Pakistani group were BMI, age, season and vitamin D supplements. Equivalent results for the Bangladeshi group results were age and season (online Supplementary Table S7). When the logistic regression model was conducted within sex, in women the following factors were predictive of vitamin D deficiency: ethnicity; BMI; age; oily fish; season and vitamin D supplements. In men, predictors were the same as women, but in addition, sun protection and vegetarianism were also predictors (online Supplementary Table S8).

## Discussion

We found that serum 25(OH)D concentration was very low among the UK Biobank South Asians: 50 % of participants had 25(OH)D less than 25 nmol/l, and 20 % of participants had 25(OH)D < 15 nmol/l which, although not a commonly used cut-off point, still represents severe vitamin D deficiency and likely osteomalacia. The sub-analysis showed that this rose to 29 % <15 nmol/l when those who had serum 25(OH)D outside the detection limit were included in the analysis, although overall median 25(OH)D only fell by 2 nmol/l. Our logistic regression modelling showed that being male, of Pakistani ethnicity, having a higher BMI, being closer to middle age (40–59 years old), never consuming oily fish, having summer sun exposure less than 5 h/d, not using a vitamin D-containing supplement, having a blood draw in winter or spring and being vegetarian were associated with increased odds of vitamin D deficiency (serum 25(OH)D < 25 nmol/l).

We can speculate as to why the Bangladeshi group had the highest 25(OH)D concentration, albeit by only a relatively small degree (7 nmol/l). This may be due to the higher oily fish intake in this group, with 52 % of Bangladeshis eating oily fish two or more times per week, compared with only 9 % of Pakistanis and 11 % of Indians. Our previous paper explored vitamin D intake in the Bangladeshi, Indian and Pakistani groups of the UK Biobank^(3)^ and found that Bangladeshis had a higher intake (3 µg/d) compared with 1·5 µg/d in Pakistanis and 1·0 µg/d in Indians. However, in our logistic regression model, in the current analysis, vitamin D intake was not included as it was not statistically significant. This means that the higher Bangladeshi intake was not necessarily associated with vitamin D deficiency once other factors were controlled for. Moreover, it is a very low intake, considering that the Scientific Advisory Committee for Nutrition^(15)^ recommends 10 µg/d to ensure a 25(OH)D of over 25 nmol/l without the need for significant summer sun exposure.

In terms of the drivers of 25(OH)D deficiency in the three ethnic sub-groups, the logistic regression sub-analyses in the Pakistani and Bangladeshis groups suggest that age was a predictor, with those over 60 years having a reduced odds of 25(OH)D deficiency. This suggests that in these groups the focus needs to be on middle-aged individuals who are likely to be in part-time or full-time work and may get little sun exposure, or have other factors that reduce 25(OH)D concentration, as a result. In the Indian and Pakistani groups, BMI was an important predictor of 25(OH)D deficiency. Therefore, in these two groups, reduction in BMI to a healthy body weight could be beneficial for reducing odds of deficiency. Increased oily fish consumption was a predictor in the Indian group, so this could be a factor to focus on (in those who eat fish). In the Pakistani group, increased vitamin D supplementation was a predictor and so this is an important focus for this group. Finally, in the Indian group, overall sunlight exposure was a predictor of 25(OH)D deficiency and so increasing sun exposure in this group could be beneficial in terms of reducing odds of deficiency.

We must bear in mind that the Bangladeshi group had a much smaller sample size than the other two ethnic sub-groups in the logistic regression model (*n* 171). Bangladeshis were more likely to be second-generation migrants than were the other two groups, although it is unclear as to what impact this would have on 25(OH)D concentration. Finally, it is likely that the Indian group had a slightly inflated 25(OH)D concentration as there were slightly more Indians than Bangladeshis or Pakistanis having blood draws in the summer.

Although the regression model highlighted that being male was associated with an increased odds of deficiency, the actual difference in 25(OH)D concentration between males and females was very small (around 2 nmol/l). There were meaningful sex differences in BMI, oily fish consumption, vegetarianism, usage of vitamin D supplements, time spent outdoors and usage of sun protection. These were also significant predictors in the logistic regression models. Therefore, public health interventions to improve vitamin D status in women could include initiatives to reduce obesity, increase oily fish consumption and more time spent outdoors in summer. Similarly, 20 % of the men were obese, 25 % ate no oily fish and 9 % spent <1 h a day outdoors in the summer, meaning similar interventions may also be useful for men. In men, there was the problem of low vitamin D containing supplement use (80 % did not use a supplement), which could also be targeted by public health interventions. The logistic regression sub-analysis supported the above observations, suggesting that BMI, age, oily fish, season and vitamin D supplements were predictors of deficiency in women. The predictors in men were the same, but with the addition of sun protection non-usage and being vegetarian.

When assessing age, it was unexpected that the slightly younger subset (40–59 years old) would have an increased odds of deficiency than those who were older (60 years and over). This is because it is usually assumed that older persons are more at risk of deficiency than younger persons due to a combination of a reduced ability of the skin to synthesise 25(OH)D with ageing^(22)^ as well as a possibly reduced sun exposure as people become more frail and spend less time outdoors. It is likely that those in this sample were not old enough to be showing these detrimental ageing effects. Indeed, of the 6433 participants, 95 % of participants were aged 67 years or younger. It is likely that had the participants been older and frailer, we would have seen a reduction in 25(OH)D with age. In the younger old (i.e. those in their 60s and 70s), who are still relatively healthy, it could be hypothesised that retirement may actually pose an opportunity for increased sunlight exposure (e.g. due to more leisure time). In contrast, persons closer to middle age are more likely to be working full time as well as possibly having significant family and community responsibilities.

It was as expected that those who were overweight or obese had a higher odds of deficiency, with those being obese having the highest odds. This is in agreement with numerous observations of a negative association between BMI and 25(OH)D status in adults and children^(23)^. It is theorised that this is due to either volumetric dilution due to an increased body size or the sequestering of 25(OH)D in adipose tissue, with more sequestering when there is a higher adipose tissue mass^(24)^. These factors suggest a higher requirement for vitamin D intake or production, to achieve the same serum 25(OH)D as a person with less adiposity, as borne out by recent supplementation trials^(25)^.

We found that those with a blood draw in summer or autumn had a higher 25(OH)D than those with blood draws in winter or spring. This concurs with the known ‘vitamin D winter’ in the UK, whereby production of 25(OH)D ceases from September/October to April/May (exact month dependent on latitude). Being vegetarian (defined as never consuming meat or fish) was associated with increased odds of vitamin D deficiency in the regression model. This is as would be expected given that the majority of the richer sources of vitamin D are from animal products. We assumed that the vegetarians ate eggs so this was included in vegetarian intake. Of note, the results of our analysis may differ if the vegetarian variable was recoded to either assume no egg intake or to allow fish intake.

We found a lower 25(OH)D by 6–7 nmol/l in Scotland compared with London. There was the expected 25(OH)D gradient with Scotland having the lowest 25(OH)D (19 nmol/l), followed by Northern England, Midlands and Wales (North and South Wales combined) at 19–20 nmol/l, with London and Southern England having the highest 25(OH)D at 24–25 nmol/l. However, the differences in 25(OH)D by region were not statistically significant in the logistic regression model when other factors were controlled for. Indeed, Scotland had a higher number of men in the sample (60 % men, 40 % women) compared with London which had an even split between men and women. This could partly explain the slightly lower 25(OH)D seen in Scotland, in addition to other factors. Previous work has also observed a north-south gradient in 25(OH)D across the UK, with Southern England (Surrey), being found in White postmenopausal women to be 20 nmol/l higher than their Aberdeen (Scotland)-dwelling counterparts^(6)^. In the latter study, these groups were homogenous for sex and ethnic group, as well as socio-economic status, so these factors could not have influenced this result.

The following variables were not statistically significant predictors of 25(OH)D deficiency: born in the UK/Republic of Ireland *v*. elsewhere (i.e. first- or second-generation migrant), use of statins, smoking, Townsend deprivation index, skin tone, sunbed/solaria usage, income, region and vitamin D intake. Of note, the variables for smoking and for sunbed/solaria usage may not have worked well in the model when trialled as the categories were very unbalanced (i.e. there were few smokers or few sunbed/solaria users). Vitamin D intake may have not been associated with 25(OH)D concentration as it was so low (1–3 µg/d). It may become a significant contributor to 25(OH)D concentration if intakes were higher, as can be seen by the statistically significant association that supplement use has with 25(OH)D concentration, whereby the vitamin D dose is larger (10 µg/d or higher).

Overall, this analysis suggests that there is an urgent need for public health interventions to prevent and treat vitamin D deficiency in UK South Asians. As a consequence, reducing vitamin D deficiency will help reduce rates of non-communicable diseases in this population group. We have found that although 25(OH)D concentrations are relatively similar among the ethnic and sex groups studied here, there may be slightly different drivers of deficiency in each of these groups that need to be explored further.

### Strengths and limitations

To the authors’ knowledge, this is the largest analysis to date of 25(OH)D status in European-dwelling South Asians. Of concern, in terms of generalisability, these results may underestimate the true extent of vitamin D deficiency in this population as the UK Biobank participants may be healthier and more educated than the general population. The Welsh and Scottish data are particularly novel as have not been presented before, at least for a specific group of South Asian population (rather than a mixed Asian group).

The Bangladeshi group was relatively small compared with the other two ethnic groups, with 207 in the descriptive analysis and 171 in the logistic regression model. This may mean that they are more likely to be biased in some way which may explain why their 25(OH)D concentration was higher than the other two groups. However, the mean and trimmed mean were similar in all ethnic groups (no difference larger than 1·6 nmol/l), suggesting no pull of extreme high or low values on the result.

Women were slightly overrepresented in the group that had data outside of detectable limits, compared with original percentages of women in the 8024 cohort, and this may partly explain why men had a very slightly lower 25(OH)D than women (more women had been excluded due to having very low values). Regarding the assay methodology, as discussed previously, the use of the DiaSorin Liaison XL assay may lead to some bias in the results as this generally underestimates total 25(OH)D as compared with the ‘gold standard’ method (LC–MS)^(13)^. A more effective method for detecting low 25(OH)D values is suggested for future assessments in South Asian individuals, due to the very low 25(OHD) concentrations observed.

### Conclusion

To conclude, severe deficiency in the UK Biobank South Asians was much higher than would be expected, with 20 % having 25(OH)D concentration <15 nmol/l (very severe deficiency), 55 % had 25(OH)D < 25 nmol/l (severe deficiency) and 92 % had 25(OH)D < 50 nmol/l (insufficiency). This suggests a clear vitamin D deficiency epidemic in UK-dwelling South Asians. Predictors of serum 25(OH)D < 25 nmol/l included being male, of Pakistani ethnicity, having a higher BMI, being 40–59 years old, never consuming oily fish, having summer sun exposure <5 h/d, not using a vitamin D-containing supplement, having a measurement in winter or spring and being vegetarian. Our analyses suggest that there is an urgent need for public health interventions to prevent and treat vitamin D deficiency in UK South Asians. Although 25(OH)D concentrations are relatively similar among the ethnic and sex groups studied, there may be slightly different drivers of deficiency in each of these groups.

## Supplementary material

For supplementary material accompanying this paper visit http://dx.doi.org/10.1017/S0007114520002779. click here to view supplementary material
