# Supplementary material for: Very high prevalence of 25-hydroxyvitamin D deficiency in 6433 UK South Asian adults: analysis of the UK Biobank Cohort
Source: Br J Nutr. 2020 Jul 22;125(4):448–59. doi: 10.1017/S0007114520002779 (PMC7844605; doi:10.1017/S0007114520002779)
Supplement: Supplementary file 1 [file S0007114520002779sup001.pdf]

Supplementary File

Contents

Variable coding 2

Non-dietary continuous variables 2

General information on Non-Dietary Categorical Variables 2

Re-coding of Non-Dietary Categorical variables 3

Missing variables 4

Sub-analysis 4

Primary outcomes when including those under the detection limit 4

Table S1: Median (IQR) for 25(OH)D, as well as % of participants below 25(OH)D cut-offs by group and gender when those outside of limits are included (n 7257) 6

Table S2: 25(OH)D by season- including those under detection limit (n7257) 7

Table S3: 25(OH)D by region- including those under detection limit (n7257) 8

Table S4 Descriptives % (those who have a vitamin D measurement- n6433)- Main analysis .. 9

Table S5: 25(OH)D concentration by season, gender and ethnicity- Main analysis (n 6433) .. 11

Table S6: 25(OH)D by region and gender- Main analysis (n 6433) 12

Table S7: Odds of having <25nmol/L concentration: Logistic regression model including a variety of demographic, anthropometric, dietary and lifestyle related variables-*split by ethnic group* 14

Table S8: Odds of having <25nmol/L concentration: Logistic regression model including a variety of demographic, anthropometric, dietary and lifestyle related variables-*split by gender* 16

Figure S1 Serum 25(OH)D by gender (n6433, main analysis) 19

Figure S2 % within each 25(OH)D cut-off category by gender- average for the year (all data combined)(n 6433, main analysis) 20

Supplementary File

Variable coding

Non-dietary continuous variables

Fasting time was self-reported by participants at time of blood draw and was calculated as the time since last meal or drink (excluding plain water). Fasting time and Townsend deprivation index were retained as continuous variables as it was not deemed necessary to categorise these. To assess the impact of older-age vs. middle-age on 25(OH)D concentration, the age variable (in number of years) was re-coded into 2 categories (40-59 and ≥ 60 years old).

Body Mass Index (BMI) and waist: hip ratio were calculated by Biobank staff from measured body weight and height and waist/hip measurements respectively, when the participant underwent body composition assessment. In order to increase clinical relevance, usual clinical cut-offs were used to code BMI into a categorical variable. The following cut-offs were used: ≤25.4 kg/m^2 Normal/Underweight; 26-29.4 kg/m^2 overweight and 30 kg/m^2 or higher obese. Underweight (BMI ≤17.4 kg/m^2) was not classified as a separate category as only few participants met this definition. Finally, daily time spent outdoors in summer was reported by the participant in hours. In order to overcome the issue of less than 1h per day being coded originally as -10, the whole variable was re-coded from a continuous variable to a categorical variable with the following categories: ≤1h per day; 1-2h per day; 3-4h per day and ≥5 hours per day.

General information on Non-Dietary Categorical Variables

All categorical variables listed here were self-reported, unless otherwise stated. Most questions were completed via a touchscreen computer based questionnaire (ethnicity, gross household income, immigration year, self-reported health, smoking status, skin tone, use of sun protection, use of solarium/sunbed lamps, statin and contraceptive pill use). A full list of the touchscreen questions can be found at the UK Biobank website: http://biobank.ndph.ox.ac.uk/showcase/showcase/docs/TouchscreenQuestionsMainFinal.pdf . Some questions were asked at the assessment centre reception at recruitment via a questionnaire (sex, assessment centre attended), or via a face to face interview at the baseline assessment (country of birth). Assessment centre attended, date and time of blood sample was reported by staff. Statin use was inferred from a positive answer to self-reported use of cholesterol lowering medications (there was no specific question using the word 'statins').

Re-coding of Non-Dietary Categorical variables

Ethnicity was not recoded, being retained as Bangladeshi, Indian and Pakistani. The time of day variable was coded from the actual time stamp (h/min/sec) into to the following categories for ease of analysis: 8:00 to 12:59, 13:00 to 15:59 and 16:00 to 21:59. There were no blood draws from 22:00 to 07:59. For season, the month was extracted from the blood draw date-time stamp and was re-coded as follows: Winter (December-February), Spring (March-May), Summer (June-August), Autumn (September to November).

Gross household income was recoded from the initial 5 categories coded in the data (<£18K, £18-30.9K, £31-51.9K, £52-100K, >£100K) to 4 categories ((<£18K, £18-30.9K, £31-51.9K, ≥ 52K). This was because the final category (>100K) had only a relatively small number of participants compared with the others. Assessment centre attended, a proxy for general area of residence, was recoded into 6 categories according to the 9 official regions of England (excluding the East of England where there were no assessment centres). The English region categories were then collapsed into 4 categories: Northern England (North-West, North-East, Yorkshire and the Humber); Southern England (South-East); Greater London and the Midlands (East Midlands and West Midlands) to make comparisons meaningful. Scottish and Welsh centres were coded as Scotland and Wales respectively.

For country born (UK/Republic of Ireland (ROI) vs elsewhere), a new dummy variable was produced with all positive responses across England, Wales, Scotland, Northern Ireland and Republic of Ireland vs. those who selected elsewhere. A new immigration decade variable was created from the continuous variable for year (given as actual year e.g. 2003), by recoding the continuous variable as up to 1959, 1960-1979, 1980-1999, 2000 or later.

Self-reported health was recoded from 4 categories (excellent, good, fair, and poor) into 2 categories (good/excellent vs. fair/poor). A new dummy variable was created for both oral contraceptive usage (Females only, coded as Yes/No) and statin use (Males and Females, coded as Yes/No) based on variables where participants chose a yes or no for current use of each of these. The smoking status variable was recoded from 3 categories (never, previous and current) to 2 categories (current vs. never/previous).

Skin tone was recoded from 6 categories (very fair/fair, light olive/dark olive, brown/black) to 3 categories (very fair/fair, light olive/dark olive, brown/black) in order to ensure balanced categories in terms of participant numbers. Use of sun protection was recoded from 5 categories (never/rarely, sometimes, most of the time, always, don’t go out in sunshine) to 3 categories

(Never/rarely, sometimes, most of time/always). The ‘don’t go out in sunshine category’ was removed as it only contained a very small number of participants and also did not relate to sun protection use. Finally, use of solarium lamps (hours) was recoded from a continuous variable, whereby 90% of values were zero, into a dummy variable yes (any use) vs. no (zero use).

Missing variables

To assess reason for missing vitamin D measurement, the reporting of the assay variable was used. Both the aliquot problem categories (unrecoverable aliquot problems and aliquot 4 used) were collapsed into one aliquot variable ‘aliquot related problem’ (n=49) due to low counts for these individual categories. All other categories remained as originally coded (i.e. no data returned, original value above or below reportable limit, analyser deemed result not reportable for reason other than above or below reportable range). *Note: ‘no data returned’ was a valid reason given by UK Biobank.* In terms of reason for a missing blood draw, when assessing the reason for no blood draw, variables that coded one reason were examined as were those including multiple reasons per person to use a much information as possible to inform the reason. The above information was used to inform the information given in Figure 1.

Sub-analysis

Primary outcomes when including those under the detection limit

Results are given as median (Interquartile Range, IQR) unless otherwise stated. The proportion of persons with 25(OH)D<15 nmol/L (very severe deficiency) was very high at 29%. In addition, nearly all participants had 25(OH)D <75 nmol/L (Table S1). In terms of ethnic sub-groups, Pakistanis and Indians had a lower median 25(OH)D (18 to 22 nmol/L) compared with Bangladeshis (26 nmol/L). However, this ethnic difference in median 25(OH)D is likely only of minimal biological relevance. Of note, Bangladeshis did have much lower proportion of people below 15nmol/L than did Indians or Pakistanis. Men had a slightly lower 25(OH)D (20 (17) nmol/L) than did women (22 (21) nmol/L), but as with the ethnic difference this was likely of minimal biological relevance. The IQR is very wide for both sexes, suggesting large variability in 25(OH)D between individuals.

A two way between subjects ANOVA showed a gender x season interaction (P<0.001) but no ethnicity x season interaction (P=0.44). Main effects for both ethnicity and season were statistically significant (P<0.001). Of note, although statistically significant, the interaction

between season and gender would not be considered biologically meaningful, as there was only a 0.5 to 2.5 nmol/L gender difference by season (Table S2).

A Kruskal Wallis test showed a statistically significant difference in 25(OH)D status between regions, with a latitude gradient present (Scotland lowest at a median (IQR) of 16 (16) nmol/L and London highest at 24 (20) nmol/L, Table S3). There was a clear latitude gradient seen in the men (Scotland 16 nmol/L, Northern England, Midlands and Wales 17-19 nmol/L and London and Southern England 22 nmol/L; $P<0.001$). The gradient was present in women but Scotland was similar to Northern England (Scotland, Northern England, Midlands and Wales 17-18 nmol/L, and London and Southern England 22-25 nmol/L, $P<0.001$).

Table S1: Median (IQR) for 25(OH)D, as well as % of participants below 25(OH)D cut-offs by group and gender when those outside of limits are included (n 7257)

Group/25(OH)D nmol/L	n	Median (nmol/L)	IQR (nmol/L)	<15 nmol/L	<25 nmol/L	<50 nmol/L	<75 nmol/L	P value for medians*
All Biobank South Asian	7257	20.7	18.5	28.7	60.0	92.4	99.1	-
Bangladeshi	212	25.8	15.0	9.0	44.8	91.5	100.0	P<0.001 ethnic
Indian	5395	21.6	19.6	27.5	57.4	91.5	99.0	
Pakistani	1650	17.6	14.7	35.4	70.2	95.6	99.4	
South Asian Men	3922	20.1	16.7	29.5	62.9	94.5	99.5	P<0.001 gender
South Asian Women	3335	21.8	21.1	27.9	56.5	90.0	98.7	

IQR, Interquartile Range. *=p value: Mann Whitney for South Asian men vs. South Asian women. Kruskal Wallis test for Bangladeshi vs Indian vs Pakistani.

Table S2: 25(OH)D by season- including those under detection limit (n7257)

	Spring			Summer			Autumn			Winter			
**Group/25(OH)D nmol/L**	**Median (nmol/L)**	**IQR (nmol/L)**	**n**	**Median (nmol/L)**	**IQR (nmol/L)**	**n**	**Median (nmol/L)**	**IQR (nmol/L)**	**n**	**Median (nmol/L)**	**IQR (nmol/L)**	**n**	**P value for medians***
All South Asian	18.2	16.8	1864	24.8	18.5	2371	21.1	18.9	1714	16.1	15.6	1308	-
Bangladeshi	24.0	14.0	54	29.0	13.4	58	26.5	12.0	49	23.0	20.1	51	Ethnic x Season =0.44 Ethnic main effect P<0.001 Season main effect P<0.001
Indian	18.8	18.0	1364	25.3	19.0	1845	22.2	20.4	1291	17.0	17.6	895	
Pakistani	16.3	14.0	446	22.2	16.5	468	18.1	14.1	374	14.3	9.8	362	
South Asian Men	17.8	14.7	1025	25.1	17.1	1240	20.4	16.8	921	15.4	12.9	736	Gender x Season P =0.001
South Asian Women	19.5	20.9	839	24.6	19.6	1131	22.9	21.4	793	16.9	18.9	572	

IQR, Interquartile Range. *P value from two-way ANOVA

Table S3: 25(OH)D by region- including those under detection limit (n7257)

	**All SA**			**Men**			**Women**			
**25(OH)D**	**Median**	**IQR**	**n**	**Median**	**IQR**	**n**	**Median**	**IQR**	**n**	**Assessment Centre/Latitude**
Scotland	16.4	16.0	170	15.5	15.8	102	18.3	21.0	68	Glasgow and Edinburgh<br>55.9°N to 56.0°N
Northern England	18.2	16.0	1665	18.5	14.5	981	17.7	19.0	684	Manchester, Stockport, Bury, Leeds, Liverpool, Middlesbrough, Newcastle, Sheffield, Cheadle<br>53.4°N to 55 °N
Midlands	18.1	16.8	1495	18.1	15.3	848	18.1	19.6	647	Birmingham, Nottingham, Stoke<br>52.5 °N to 53 °N
Wales	16.7	16.5	111	17.1	16.7	63	16.7	17.4	48	Cardiff, Swansea, Wrexham<br>51.5°N to 53.0°N
Southern England	21.8	19.7	566	21.7	17.9	289	22.2	21.4	277	Bristol, Oxford and Reading<br>51.5°N to 51.8°N
London	23.5	19.9	3250	22.3	18.1	1639	25.0	22.2	1611	Bart's, Croydon and Hounslow<br>51.4°N to 51.5°N
P value*	<0.001<br>All South Asians			<0.001 Men only			<0.001 Women only			

IQR, Interquartile Range . *P value based on Kruskal Wallis test

Table S4 Descriptives % (those who have a vitamin D measurement- n6433)- Main analysis

		All SA<br>n 6433*	Bangladeshi<br>n 207	Indian<br>n 4792	Pakistani<br>n 1434	Men<br>n 3506	Women<br>n 2927
**Time of day blood draw %**	**8:00 to 12:59**	28.7	26.6	29.2	27.7	29.8	27.5
	**13:00 to 15:59**	31.4	37.2	31.3	31.1	29.7	33.5
	**16:00 to 21:59**	39.7	36.2	39.5	41.2	40.4	39.0
	**Missing**	0.1	-	-	-	-	-
	**P value**	-	P=0.29 ethnic			P=0.004 gender	
**Immigration decade† %**	**Up to 1959**	2.0	0.5	2.7	1.5	2.1	2.7
	**1960-1979**	61.0	45.4	74.1	61.9	69.7	71.6
	**1980-1999**	16.3	43.8	16.1	24.9	18.5	19.3
	**2000 or later**	7.1	10.3	7.2	11.6	9.7	6.4
	**Missing as Born UK/ROI**	10.6	-	-	-	-	
	**Other Missing**	3.0	-	-	-	-	-
	**P value**	-	P<0.001			P<0.001	

		All SA<br>n 6433*	Bangladeshi<br>n 207	Indian<br>n 4792	Pakistani<br>n 1434	Men<br>n 3506	Women<br>n 2927
**Use of solarium/sunbed lamps %**	**Not used**	85.3	94.5	96.7	95.3	96.0	96.8
	**Less than once per year**	1.8	3.4	1.9	2.3	2.4	1.6
	**Once or more per year**	1.4	2.1	1.4	2.4	1.6	1.7
	**Missing**	11.4					
	**P value**		P=0.08 ethnic			P=0.10 gender	

SA=South Asian, all p values are chi-square. *Percent including missing data, all other percentages are valid percent (i.e excluding missing data). † n 5616 that immigrated to UK/ROI.

Table S5: 25(OH)D concentration by season, gender and ethnicity- Main analysis (n 6433)*

	**Spring**			**Summer**			**Autumn**			**Winter**			
**Group/25(OH)D nmol/L***	**Median (nmol/L)**	**IQR (nmol/L)**	**n**	**Median (nmol/L)**	**IQR (nmol/L)**	**n**	**Median (nmol/L)**	**IQR (nmol/L)**	**n**	**Median (nmol/L)**	**IQR (nmol/L)**	**n**	**P value for medians†‡**
All South Asian	20.7	17.1	1586	25.8	18.1	2255	23.0	18.8	1549	19.5	15.6	1043	
Bangladeshi	24.0	13.6	53	29.2	12.9	56	26.7	11.9	48	23.4	21.6	50	Ethnic x Season P=0.13. Ethnic main effect P<0.0001 Season main effect P<0.0001
Indian	21.4	18.3	1159	26.2	18.9	1755	23.8	19.8	1165	20.9	18.0	713	
Pakistani	18.0	14.0	374	23.3	16.3	444	19.0	14.6	336	16.1	8.8	280	
South Asian Men	19.4	13.9	875	25.6	16.7	1191	21.6	16.7	841	18.1	13.5	599	Gender x Season P<0.0001
South Asian Women	22.7	21.4	711	25.9	19.3	1064	24.5	20.9	708	21.7	19.3	444	

IQR, Interquartile Range. *Seasonal data is NOT repeated measures †P value is two way between groups Anova to see the association between ethnic group (or gender) and season on 25(OH)D. ‡Log 25(OH)D used for analysis.

Table S6: 25(OH)D by region and gender- Main analysis (n 6433)

	All South Asians			Men			Women			
**25(OH)D nmol/L**	**Median**	**IQR**	**n**	**Median**	**IQR**	**n**	**Median**	**IQR**	**n**	**Latitude***
Scotland	18.7	16.6	142	16.7	15.7	86	20.9	20.6	56	Glasgow and Edinburgh<br>55.9ºN to 56.0ºN
Northern England	20.3	16.1	1430	20.1	14.6	856	20.7	18.6	574	Manchester, Stockport, Bury, Leeds, Liverpool, Middlesbrough, Newcastle, Sheffield, Cheadle<br>53.4ºN to 55 ºN
Midlands	20.4	17.0	1280	20.2	14.9	739	20.9	18.3	541	Birmingham, Nottingham, Stoke<br>52.5 ºN to 53 ºN
Wales	19.1	16.0	89	20.0	16.7	51	18.8	16.5	38	Cardiff, Swansea, Wrexham<br>51.5ºN to 53.0ºN
Southern England	23.8	19.3	514	22.7	18.6	269	24.7	20.7	245	Bristol, Oxford and Reading<br>51.5ºN to 51.8ºN
London	25.0	19.6	2978	23.7	17.2	1505	26.5	21.7	1473	Bart's, Croydon and Hounslow<br>51.4ºN to 51.5ºN
P value†	<0.001All South Asians			<0.001 Men only			<0.001 Women only			

*IQR, Interquartile Range. *Based on location of assessment centres. †Kruskal Wallis test*

Table S7: Odds of having <25nmol/L concentration: Logistic regression model including a variety of demographic, anthropometric, dietary and lifestyle related variables-*split by ethnic group*

	Bangladeshi n 146				Indian n 3222				Pakistani n 1072			
	n	**OR†**	**Lower 95% CI**	**Upper 95% CI**	***n***	**OR†**	**Lower 95% CI**	**Upper 95% CI**	***n***	**OR†**	**Lower 95% CI**	**Upper 95% CI**
**Sex**												
Female	43	1.00			1598	1.00			398	1.00		
Male	103	1.04	0.45	2.43	1624	1.38	1.18	1.62	674	0.96	0.71	1.30
**Body Mass Index‡**												
≤25.4	58	1.00			1164	1.00			262	1.00		
26-29.4	65	1.09	0.47	2.54	1440	1.32	1.12	1.56	502	1.40	1.00	1.96
≥30	23	1.28	0.41	4.04	618	1.56	1.27	1.93	308	1.39	0.96	2.03
**Age**												
40-59 years old	112	1.00			2181	1.00			846	1.00		
60 years and over	34	0.17	0.06	0.50	1041	0.59	0.51	0.69	226	0.53	0.39	0.74
**Oily Fish Consumption**												
Never	7	1.00			1366	1.00			217	1.00		
<Once per wk	28	0.73	0.10	5.52	769	0.93	0.73	1.19	430	1.17	0.80	1.69
Once per wk	40	0.93	0.12	6.98	794	0.87	0.68	1.11	334	0.97	0.66	1.44
2 or more times per wk	71	0.96	0.14	6.77	293	0.50	0.36	0.68	91	0.90	0.52	1.55
**Daily summer sun**												
<1h	21	1.00			374	1.00			109	1.00		
1-2h	57	0.68	0.21	2.16	1328	0.81	0.63	1.04	424	1.16	0.72	1.88
3-4h	42	0.63	0.17	2.27	894	0.79	0.61	1.03	271	0.87	0.56	1.45
≥5h	26	0.43	0.11	1.79	626	0.72	0.55	0.95	268	0.91	0.54	1.53
**Season of blood draw**												
Spring	36	1.00			765	1.00			292	1.00		

	Bangladeshi n 146				Indian n 3222				Pakistani n 1072			
	n	**OR†**	**Lower 95% CI**	**Upper 95% CI**	***n***	**OR†**	**Lower 95% CI**	**Upper 95% CI**	***n***	**OR†**	**Lower 95% CI**	**Upper 95% CI**
Summer	39	0.26	0.09	0.77	1231	0.50	0.41	0.61	349	0.43	0.30	0.61
Autumn	32	0.25	0.08	0.81	774	0.72	0.58	0.89	224	0.83	0.56	1.23
Winter	39	0.90	0.31	2.61	452	0.98	0.76	1.25	207	1.49	0.97	2.31
**Sun protection usage§**												
Never/rarely	93	1.00			1452	1.00			646	1.00		
Used sometimes	41	0.62	0.26	1.45	1211	0.70	0.60	0.83	302	0.92	0.68	1.26
Used most of time/always	12	0.88	0.21	3.69	559	0.83	0.67	1.03	124	0.82	0.52	1.27
**Vitamin D supplement‖**												
User	23	1.00			890	1.00			232	1.00		
Non-user	123	2.92	0.92	9.25	2332	2.72	2.30	3.22	840	4.36	3.15	6.04
**Vegetarian**												
Yes	1				900	1.00			4	1.00		
No	145	-	-	-	2322	0.78	0.61	1.00	1068	3.18	0.30	34.0
**Constant**		0.00				1.24				0.23		

Wk=week. †OR= odds of having serum 25(OH)D <25nmol/L (≥25nmol/L=reference); ‡kg/m^2, §= usage of sunscreen lotion or hat, ‖ vitamin D containing supplement (i.e. single supplement, or multivitamin). Note: Vegetarian variable for Bangladeshis gives abnormal value due to only n=1 vegetarian in this group

Table S8: Odds of having <25nmol/L concentration: Logistic regression model including a variety of demographic, anthropometric, dietary and lifestyle related variables-*split by gender*

	Female n 2039				Male n 2401			
	n	**OR†**	**Lower 95% CI**	**Upper 95% CI**	***n***	**OR†**	**Lower 95% CI**	**Upper 95% CI**
**Ethnicity**								
Indian	1598	1.00			1624	1.00		
Pakistani	398	1.57	1.21	2.02	674	1.27	1.03	1.56
Bangladeshi	43	0.78	0.41	1.52	103	0.49	0.31	0.76
**Body Mass Index**‡								
≤25.4	691	1.00			793	1.00		
26-29.4	848	1.45	1.17	1.79	1159	1.22	1.00	1.49
≥30	500	1.47	1.15	1.88	449	1.60	1.24	2.08
**Age**								
40-59 years old	1482	1.00			1657	1.00		
60 years and over	557	0.58	0.47	0.71	744	0.54	0.45	0.65
**Oily Fish Consumption**								
Never	878	1.00			712	1.00		
<Once per wk	489	1.05	0.78	1.43	738	0.95	0.72	1.25
Once per wk	500	0.86	0.63	1.16	668	0.94	0.71	1.24
2 or more times per wk	172	0.50	0.33	0.75	283	0.69	0.49	0.98
**Daily summer sun**								
<1h	311	1.00			193	1.00		
1-2h	889	0.83	0.63	1.09	920	0.96	0.68	1.37
3-4h	536	0.78	0.58	1.05	671	0.88	0.62	1.27
≥5h	303	0.78	0.55	1.09	617	0.81	0.56	1.16
**Season of blood draw**								

	Female n 2039				Male n 2401			
	n	**OR†**	**Lower 95% CI**	**Upper 95% CI**	***n***	**OR†**	**Lower 95% CI**	**Upper 95% CI**
Spring	492	1.00			601	1.00		
Summer	790	0.70	0.55	0.89	829	0.35	0.27	0.44
Autumn	474	0.86	0.66	1.13	556	0.62	0.48	0.81
Winter	283	1.07	0.78	1.46	415	1.09	0.82	1.46
**Sun protection usage§**								
Never/rarely	809	1.00			1382	1.00		
Used sometimes	769	0.82	0.67	1.02	785	0.66	0.54	0.80
Used most of time/always	461	0.89	0.70	1.14	234	0.79	0.58	1.06
**Vitamin D supplement‖**								
User	665	1.00			480	1.00		
Non-user	1374	2.84	2.32	3.46	1921	3.25	2.60	4.05
**Vegetarian**								
Yes	574	1.00			331	1.00		
No	1465	0.84	0.62	1.13	2070	0.62	0.44	0.86
**Constant**		0.84				1.95		

Wk=week. †OR= odds of having serum 25(OH)D <25nmol/L (≥25nmol/L=reference); ‡kg/m^2, §= usage of sunscreen lotion or hat, ‖ vitamin D containing supplement (i.e. single supplement, or multivitamin).

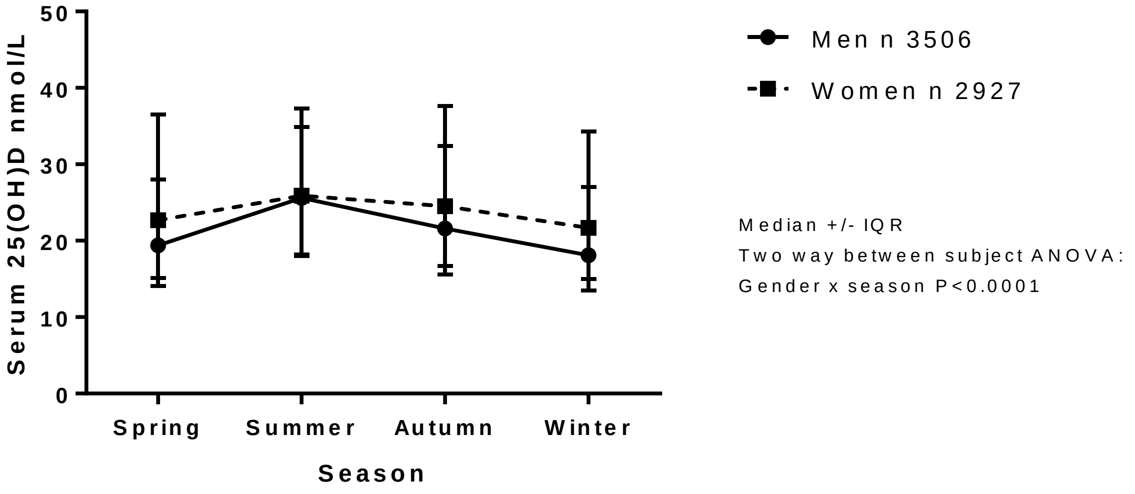

Figure S1 Serum 25(OH)D by gender (n6433, main analysis)

Each person has one measurement in one season only (data are not repeated measures). According to a two-way between subjects ANOVA there was a statistically significant interaction between gender and season, with the women having the highest 25(OH)D in each season except summer whereby it was similar to the men. Both groups were slightly higher in summer and autumn and slightly lower in winter and spring.

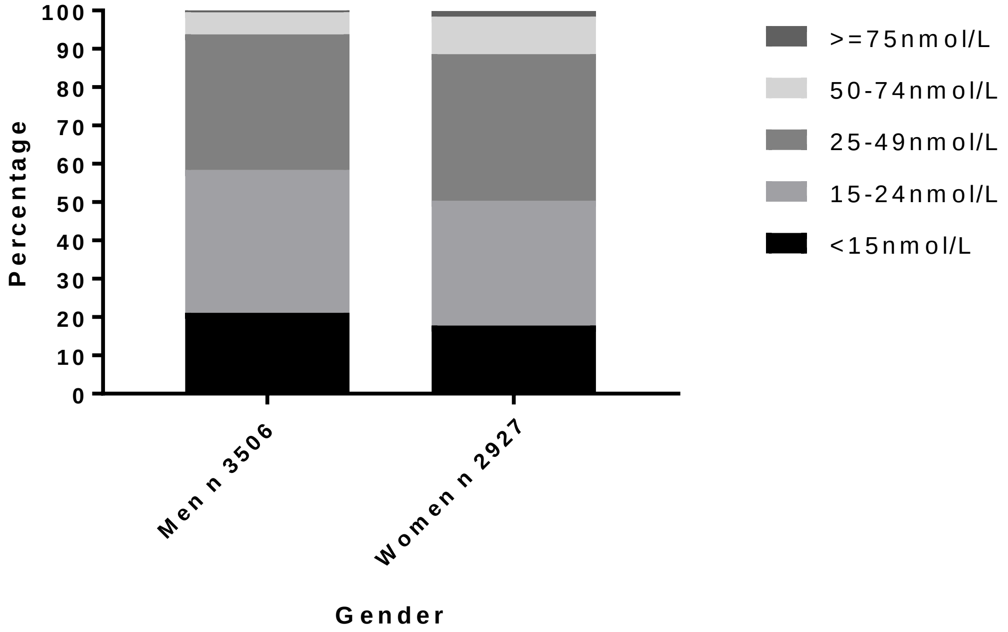

Figure S2 % within each 25(OH)D cut-off category by gender- average for the year (all data combined)(n 6433, main analysis)
